# Draft Genome Sequence of Marine Cyanobacterium *Synechococcus* sp. Strain NKBG042902, Which Harbors a Homogeneous Plasmid Available for Metabolic Engineering

**DOI:** 10.1128/genomeA.00704-14

**Published:** 2014-07-24

**Authors:** Toru Honda, Yue Liang, Daichi Arai, Yasuhito Ito, Tomoko Yoshino, Tsuyoshi Tanaka

**Affiliations:** a Division of Biotechnology and Life Science, Institute of Engineering, Tokyo University of Agriculture and Technology, Koganei, Tokyo, Japan; b JST, CREST, Koganei, Tokyo, Japan

## Abstract

The marine cyanobacterium *Synechococcus* sp. strain NKBG042902 was isolated from coastal areas in Japan. Strain NKBG042902 has four plasmids: pSY8, pSY9, pSY10, and pSY11. Moreover, the hybrid plasmid pUSY02 containing pSY11 and *Escherichia coli* plasmid pUC18 was constructed for this strain. The genetic manipulation technique using pUSY02 was established for this strain and used in metabolic engineering. Here, we report the draft genome sequence of this strain, which has 77 contigs comprising a total length of 3,319,479 bp, with a G+C content of 49.4%.

## GENOME ANNOUNCEMENT

Cyanobacteria are photosynthetic prokaryotic microorganisms that are ubiquitous in marine, freshwater, and land systems. Various useful natural compounds have been discovered and produced from cyanobacteria (1). Recently, cyanobacteria have been accepted as a promising candidate for sustainable bioenergy generation because their metabolic pathway can be modified for the production of biofuel compounds through genetic engineering (2, 3). Various biofuel compounds (e.g., 1-butanol, alkane, and ethylene) have successfully been produced from the cyanobacteria *Synechocystis* sp. PCC6803 and *Synechococcus elongates* PCC7942 (4–6), which highlights the possibility of applying the cyanobacteria for biofuel productions.

*Synechococcus* sp. strain NKBG042902 was isolated as a fast-growing cyanobacterium from the coastal areas in Nagasaki, Japan (7), and it can survive under a wide range of salt concentrations, from 0 to 5%. A hot-water extract from the NKBG042902 strain showed the ability to promote plantlet formation from somatic embryos of carrot (8). Moreover, strain NKBG042902 has four endogenous plasmids: pSY8, pSY9, pSY10 (2.6 kbp), and pSY11 (2.3 kbp) (9). The hybrid plasmid pUSY02 containing pSY11 and *Escherichia coli* plasmid pUC18 was constructed for genetic manipulation of this strain and has been successfully applied for the production of eicosapentaenoic acid (EPA) by the expression of the EPA synthesis gene cluster from a marine bacterium, *Shewanella putrefaciens* strain SCRC-2738 (10). Thus, the strain NKBG042902 can be treated as a useful host for the production of valuable compounds, which are not naturally generated by this strain. Here, we report the draft genome sequence of the marine cyanobacterium *Synechococcus* sp. strain NKBG042902.

The experimental procedure used in this study was based on a previous report (11). In general, *Synechococcus* sp. NKBG042902 was cultured in BG11 medium (ATCC catalog medium no. 617, supplemented with 3% NaCl) at 26°C under continuous illumination in 100-ml Erlenmeyer flasks placed on a reciprocating shaker. After 2 weeks of cultivation, the cells were collected and the genomic DNA was extracted using a DNeasy plant minikit (Qiagen). The sequencing of genomic DNA was performed with a Roche/454-GS Junior system, according to the manufacturer’s protocol. The reads were assembled by the software Newbler assembler version 2.5 (Roche). The draft genome sequence of strain NKBG042902 consisted of 77 contigs (>500 bp, 22.0-fold average coverage), comprising a total length of 3,319,479 bp, with a G+C content of 49.4%. The resulting contigs were annotated by the software Glimmer 3.02 and BLAST searches against a nonredundant protein sequence database of the National Center for Biotechnology Information. tRNA genes were predicted by the RNAmmer prediction server (12), and rRNA genes were predicted by the software ARAGORN version 1.2.3 (13). The draft genome sequence of the strain includes 3,412 predicted coding regions, 40 tRNA genes, and 4 rRNA genes.

### Nucleotide sequence accession numbers.

The *Synechococcus* sp. strain NKBG042902 draft genome sequence data have been deposited in DDBJ/EMBL/GenBank under accession no. BAWS00000000. The version described in this paper is version BAWS01000000.
